# Chondrosarcoma arising in monostotic fibrous dysplasia treated with total femur resection and megaprothesis: A case report

**DOI:** 10.1016/j.ijscr.2021.106194

**Published:** 2021-07-09

**Authors:** Muhammad Wahyudi, Ziad Alaztha

**Affiliations:** a Department of Orthopaedic and Traumatology, Fatmawati General Hospital, Indonesia; b Department of Orthopaedic and Traumatology, Faculty of Medicine Universitas Indonesia, Dr. Cipto Mangunkusumo Hospital, Indonesia

**Keywords:** Chondrosarcoma, Malignant transformation, Monostotic fibrous dysplasia, Total femur resection

## Abstract

**Introduction:**

Fibrous dysplasia is tumor like lesions of bone which develop as substitution of bone by an expansion of fibrous connective tissue mixed with hard trabeculae. Chondrosarcomas is one of common malignant primary bone tumor derived from heterogenous group of neoplasm producing chondroid matrix. Chondrosarcoma arising in fibrous dysplasia, especially in monostotic fibrous dysplasia is a very rare case.

**Case report:**

A 54-year-old male presented with chief complaint of pain on left thigh. Patient with history of pathological fracture on left femoral diaphysis 3 years ago due to fibrous dysplasia and had underwent curettage, open reduction, and internal fixation at other hospital. Plain radiography revealed expansive lytic lesion, interrupted periosteal reaction with plate and screw attached to the lesion, and soft tissue mass. MRI T2FS sequence showed hyperintense mass extending from subtrochanteric to distal of left femoral diaphysis. Histopathological result from biopsy suggested chondrosarcoma.

**Conclusions:**

Malignant transformation of monostotic type was less frequently compared to polyostotic type. Among all malignant transformation cases, alteration to chondrosarcoma was more scarce than other malignancy such as osteosarcoma and fibrosarcoma. Wide surgical margin and reconstruction in chondrosarcoma provide good local control and functional outcome.

## Highlights

• Chondrosarcoma arising in monostotic fibrous dysplasia is very rare.
• Wide surgical margin provides good local control, thus become the mainstay treatment of chondrosarcoma.
• Limb salvage surgery in chondrosarcoma provides good functional outcome.

## 1 Introduction

Fibrous dysplasia (FD) is a formative tumor-like disease that is characterized as an excessive expansion of fibrous connective tissue intermingled with irregular hard trabeculae by replacing ordinary bone. This disease has a lot of clinical symptoms variants due to varying degrees of mosaicism. It can be asymptomatic to severely disabling symptoms. It may occur in alone, or as a syndrome with extra skeletal disease manifestations [1]. Alteration of fibrous dysplasia to malignancy is rare with incidence in <1% of fibrous dysplasia. The most prevalence type of malignancy developed from fibrous dysplasia is osteosarcoma (70% of cases), followed by fibrosarcoma (20% of cases), and chondrosarcoma (10% of cases), with malignant fibrous histiocytoma (4% of cases) [2], [3], [4]. We reported a 54-years-old male whom initially diagnosed with monostotic fibrous dysplasia in femoral diaphysis and had transformed into chondrosarcoma after 3 years. This case report has been reported in line with the SCARE Criteria [5].

## 2 Case illustration

**Fig. 1:**
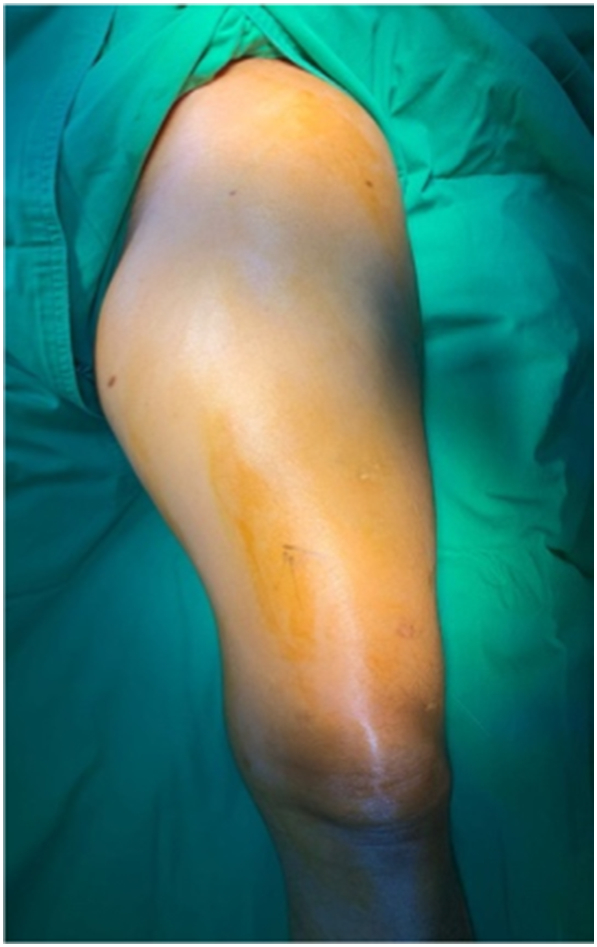
Clinical appearance of left thigh before surgery.

A 54-year-old man was referred to our institution, he complained pain on his left thigh for 2 years. The pain was felt in the midthigh region. There were no other symptoms from the anamnesis. Patient previously had pathological fracture 3 years ago on the left femoral shaft. It occurs after the patient was slipped on the bathroom and fell. The patient had undergone curettage, open reduction and internal fixation at district general hospital. Histopathological examination after first surgery suggested fibrous dysplasia. On physical examination, the lump was palpable on his postero-medial thigh. The lumps were firm and non-tender on palpation, fixated, and the size was around 14 × 8 × 11 cm (Fig. 1).

**Fig. 2:**
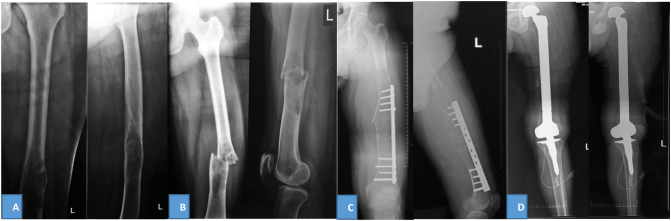
Left femur X-ray: (A) Initial X-ray of patient before fracture; (B) pathological fracture of femoral diaphysis; (C) 3 years after open reduction and internal fixation; (D) post-operative X-ray.

**Fig. 3:**
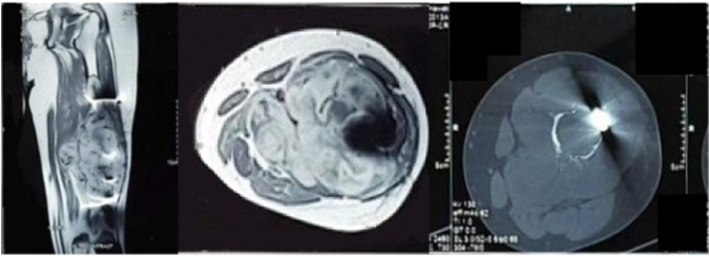
MR images showed tumor on femoral diaphysis with metal artifact.

The patient underwent diagnostical procedures, including a blood test, plain radiograph, and Magnetic Resonance Imaging (MRI). Laboratory findings were within normal ranges. Fig. 2A shows the pre-fracture condition 3 years ago, while Fig. 2B shows the fracture line in the diaphyseal of the left femur. Fig. 2C shows femoral radiograph that revealed lytic geographic lesion at femoral diaphysis, cortical disruption, interrupted periosteal reaction, soft tissue mass, and internal fixation attached to the femoral bone. Femoral MRI indicated a tumor on femoral diaphysis that measured 20 × 10 × 14 cm and displayed low to intermediate signal intensity on T1-weighted images. T2-weighted images displayed a heterogenous lesion, containing areas of low to hyperintense area. Some area showed metal artifacts due to metallic implants (Fig. 3). The histopathological result from core biopsy suggested chondrosarcoma.

**Fig. 4:**
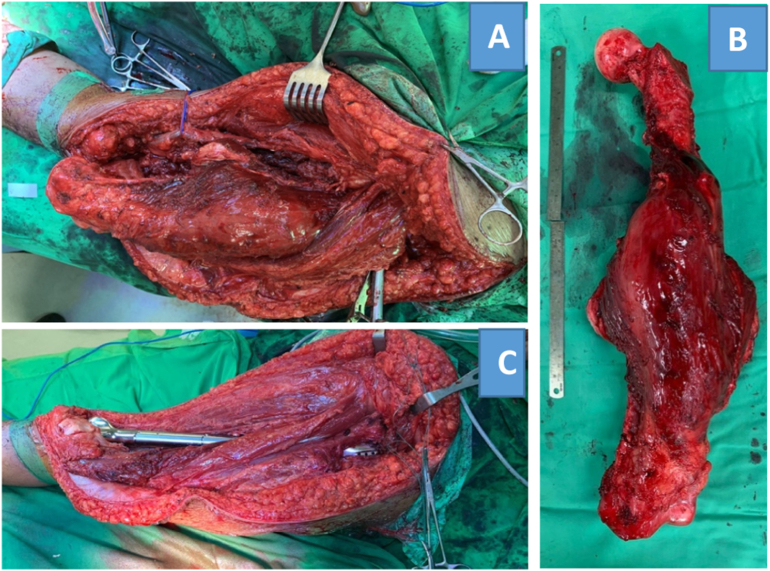
Intraoperative (A) tumor exposed; (B) resected tumor; (C) after total femur resection and reconstruction using megaprosthesis.

The patient underwent total femur resection with wide surgical margin. He was placed on supine position. The surgical incision started 4 cm proximal to the greater trochanter, it is brought curved distally to the medial thigh until anteromedial aspect of tibial tuberosity. Superficial femoral artery and vein were identified. Rectus femoris and some parts of vastus lateral muscle were preserved. The tumor was removed with wide surgical margin (Fig. 4). Reconstruction after total femur resection was done using total femur megaprothesis (Fig. 2D). The remaining hip capsule was sutured tightly with mersilene tape. Psoas muscle was tenodesed to the anterior hip capsule. External rotator muscles were sutured to the posterior hip capsule. Remaining abductor tendon is attached to the lateral aspect of the prothesis. Sartorius was sutured to the rectus femoris muscle.

The surgery took 7 h. The amount of bleeding during total femur resection was 1300 cc. There was no complication during or after surgery. We mobilized the patient with non-weight bearing and after one month of surgery we change it to partial weight bearing as tolerated. The length of stay in the hospital was 7 days.

**Fig. 5:**
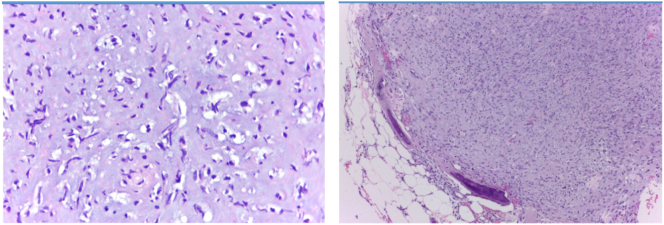
Histopathological appearance showed chondrosarcoma grade II.

The histopathology examination from the resected tumor showed cartilage matrix and hypercellular chondrocyte. The chondrocytes were varied in shape, atypical, larger in size, and hyperchromatic nuclei. There were large number of binucleation with mitosis, and same myxoid changes. The conclusion from the histopathology examination was chondrosarcoma grade II (Fig. 5).

At the 12 months follow up, the patient was in good condition. Patient had Trendelenburg gait and weak extensor knee muscles. Patient needed one crutch for walking. MSTS score evaluated functional outcomes, where the patient scored 16 or 53%. The patient did not complain of any pain. The patient also showed no sign of recurrence 12 months after the surgical procedure.

At follow up, the patient was in good condition and could work normally.

## 3 Discussion

Fibrous dysplasia results from the change from normal bone to abnormal with also obliteration of the bone marrow. The tissue in FD is rich in fibroblast-like cells. The uncontrolled GαS-mediated signaling disturbs the differentiation process of osteogenic progenitors into mature osteoblasts and osteocytes [1].

Fibrous dysplasia divided in two types, monostotic and polyostotic fibrous dysplasia. The most common, the monostotic type, affects one bone and accounts for around 70 to 85% of all cases. Moreover, the monostotic type develops later in life, usually young adults (20–30 years old), and mainly affects the ribs or the femur. Most monostotic lesions are asymptomatic and are detected incidentally in radiographic imaging. The polyostotic type, in comparison, appears earlier, typically in children younger than 10 years. The predilection of polyostotic type more often affects maxillary and other craniofacial bones, ribs, femur or tibia. Cafe-au-lait spots and hyper functional endocrine characteristic of McCune-Albright syndrome are found in 3% of polyostotic disorder, and more common in females ten times than males [6].

The clinical characteristics of fibrous dysplasia included pain, swelling, and lump. Plain radiograph of this lesion can show ground-glass lesions and osteolytic lesions and the margin can be found complete and incomplete sclerotic, mild or moderate degree of mineralization, cortical thinning or destruction, periosteal reaction and soft tissue mass. CT images were evaluated for determining location of the lesion, any related abnormality of the medullary canal, mineralization, cortical degradation, periosteal and soft tissue mass reactions. MRI abnormality was tested for any related medullary channel anomalies soft tissue edema, soft tissue mass, and contrast improvement trends [3].

Malignant degeneration can occur in fibrous dysplasia, and several cases have been published. In a study of 89 literature cases, histologic forms of malignant tumors found include osteosarcoma, fibrosarcoma and chondrosarcoma, the most common form being osteosarcoma which accounts for 60.7% of the cases [4]. Compared to the monostotic type, polyostotic type have tendency for alteration to malignant tumors [7].

Malignant transformation of fibrous dysplasia to chondrosarcoma is very rare. Prevalence of these cases was 10% of all cases [2]. There was a case series conducted by Qu et al. in 2015. This study conducted by evaluating 10 patients with monostotic fibrous dysplasia which progressed to malignant transformation. From pathological examination of all these patients, seven patients developed osteosarcoma, two patients developed fibrosarcoma, and one patient developed malignant fibrous histiocytoma. None of these patients with fibrous dysplasia developed chondrosarcoma [3].

Qu et al. stated that patients with FD and had a history of surgery should be followed up. Symptoms like cutaneous pigmentation, endocrine disturbances, and history of precocious puberty as in Mc-cune Albright syndrome or Mazabraud syndrome should be taken into consideration because this type of medical condition increasing rate for FD turned malignancy.

Radio imaging in this type of case, many literatures stated that poorly marginated, mineralized, osteolytic and cortical destruction imaging are considered as malignancy. As for the MRI showed extra osseous extent of the tumor enhanced by contrast injection [2].

In this case report, 54-year-old patient still felt pain on the left femur region after first surgery. Plain radiography revealed expansive lytic lesion, interrupted periosteal reaction with plate and screw attached to the lesion, and soft tissue mass. MRI T2FS sequence showed hyperintense mass extending from subtrochanteric to distal of left femoral diaphysis. Open biopsy revealed histopathology of the tumor was monostotic fibrous dysplasia. Three years after surgery, the patient underwent limb salvage surgery by wide excision and reconstruction using megaprosthesis. Histopathological examination from the lesion revealed grade II chondrosarcoma. The monostotic lesion underwent malignant transformation into chondrosarcoma in two years.

Treatment of this patient limb salvage surgery by wide excision and reconstruction using total femur megaprosthesis. Total femur resection was performed for diaphyseal lesions that extend proximally to the lesser trochanter and distally to the distal diaphyseal-metaphyseal junction and cause extensive bone destruction [8]. Control of the tumor and survival of patients are the primary aims of total femur resections, The aim of the prosthetic reconstruction is to restore the best possible function of the lower limb. However, several studies reported complications such [9] as mechanical complications and infections requiring hip disarticulations [10].

There was a similar case of fibrous dysplasia transformation to chondrosarcoma in pelvis region in a 10-year-old boy. This case was reported by Halawa et al. The lesion was obtained from biopsy after Kuntcher nailing and confirmed the diagnosis of fibrous dysplasia. After 23 years old, a pelvic mass appeared occupying iliac fossa and gluteal region. The mass was suspected chondrosarcoma, and the patient underwent hemipelvectomy. The diagnosis was established from histopathological examination as chondrosarcoma [11]. Malignant transformation in fibrous dysplasia is rare. Halawa et al. also described there are only 69 cases have been reported. This total includes 37 cases of osteosarcoma, 21 of fibrosarcoma and 11 of chondrosarcoma [11].

There was no sign of recurrence after 12 months of follow up. The study conducted by Halawa stated that 11 patients of chondrosarcoma in their study had such resections involving different parts of innominate bone and none of them had local recurrence or metastasis after follow-up for three to six years [11]. However, Mankin et al. in 2005 reported three cases of recurrence in 15 patients with total femur resection and 30% recurrence cases reported by Jeon et al. [10] This report is proving that long-term follow-up in chondrosarcoma transformation of fibrous dysplasia is mandatory [12].

The MSTS score in this patient was 16 or 53%. Takuya et al. study reported that the MSTS was 59% in patients with total femur resection after 52 months follow up, which mean the result in this patient was better. The Complication rate after total femur resection is high, included infection and deep vein thrombosis. In other study reported infected complication is 11% and 20% for deep vein thrombosis [9]. In this patients there was no complication reported after 12 months follow up.

## 4 Conclusion

Malignant transformation of monostotic type was less frequently compared to polyostotic type. Among all malignant transformation cases, alteration to chondrosarcoma was more scarce than other malignancy such as osteosarcoma and fibrosarcoma. Total femur resection is performed for diaphyseal lesions that extend proximally to the lesser trochanter and distally to the distal diaphyseal-metaphyseal junction and cause extensive bone destruction. Control of the tumor and survival of patients are the primary aims of total femur resections. The complication rate after total femur resection is high.

## Funding

This research did not receive any specific grant from funding agencies in the public, commercial, or not-for-profit sectors.

## Ethical approval

Ethical approval was not required in the treatment of the patient in this report.

## Consent for publication

Written informed consent was obtained from the patient for publication of this case report and accompanying images. A copy of the written consent is available for review by the Editor-in-Chief of this journal on request.

## Author contribution

Muhammad Wahyudi contributes in the study concept or design, data collection, analysis and interpretation, oversight and leadership responsibility for the research activity planning and execution, including mentorship external to the core team.

Ziad Alaztha contributes to the study concept or design, data collection and writing the paper.

## Registration of research studies

Does not need any registration.

## Guarantor

Muhammad Wahyudi, MD.

## Provenance and peer review

Not commissioned, externally peer-reviewed.

## Disclaimer

No patient or author details are included in the figures.

## Declaration of competing interest

The authors declare no conflicts of interest.
